# Sympathy in the Physician

**Published:** 1887-01-08

**Authors:** 


### Words of Consolation.

[Communications for this column should be addressed, "Chaplain," care of the Editor of The Hospital, who will gratefully welcome any suggestions or inquiries from matrons, nurses, and the staff generally.]

### XV.—Sympathy in the Physician.

St. Luke the "beloved" physician was so entitled, we believe, as much for his capacity as a sympathizer as for his skill as a practitioner. It is probable that in addition to the inspiration he received, he wrote his Gospel and the Acts of the Apostles a good deal subject to the dictation of St. Paul. Nevertheless, (such is the case with many other books in Holy Scripture) the Holy Spirit has so guided the writer as not to obscure some of the strongest and best traits in the character of the man. Probably because St. Luke had a ready faculty for sympathizing, he was chosen to exhibit the perfection of sympathy in our Blessed Lord's miracles of healing. While St. Paul, who dwells in the Epistle to the Hebrews so much on the feeling of the Great High Priest for our infirmities, must have carefully supervised the many instances recorded by St. Luke of our Lord's sympathy in action. The keynote of the third Gospel is the manifestation of the sympathy of the Great Physician. It is throughout the Gospel of the good Samaritan. At the same time, it is evident (says Bishop Wordsworth,) that this Gospel was written by one who was led and qualified by his professional studies and occupations to analyse scrupulously the characteristics of physical phenomena, especially in relation to the organization of the human frame. Ordinary diseases are carefully distinguished from one another; and they are differentiated from cases of demoniacal possession. The Medical Science of those days was crude in comparison with our own. But now that such advances have been made, is there no risk of sympathy being sacrificed to science? or if not sacrificed, is there no temptation to skilled practitioners to think that science is everything, sympathy of comparatively little consequence. A combination of exact science with sympathy is the most desirable and highest qualification of all for the physician's office. But, if it be asked which of the two is more important, we reply that, for average ministrations to suffering beings, we would much sooner have average ability with sympathy, than consummate skill or diagnosis with little or no heart. The physician who relies upon science or skill alone in dealing with a patient, however able he may be, forgets that he has to minister to a being who is "fearfully and wonderfully made," and not merely to visible flesh and blood. The person of man, strictly speaking, is composed of three parts—body, soul, and spirit. Man is material; but he has mental and spiritual faculties as well to be reckoned with. We do not think that our modern physicians are at all inclined to set aside the influence of mind on body; but we confess our fears that many of them do not sufficiently allow for the spiritual element, nor believe very firmly in the part which this element plays in the wonderful organism once fashioned in the image of God.

Those physicians who do believe in the spiritual in man—in a spiritual body of which Christ is the Head—and therefore in the influence of the Holy Spirit as exerted amongst the members of the body, are sure to be the best sympathizers. They are certain to touch chords and awaken sympathetic feelings in their patients, which materialists know nothing about. The result is that their visits are ever welcome. They are physicians "beloved" by sufferers. They inspire no fear. They are never found handling patients roughly, nor managing them as if they were merely disordered machines. Such men are not called to strictly spiritual work, and yet from their nature their ministrations become so; for they approach the weary and heavy-laden in the spirit of the Master-healer, being touched with a feeling of their infirmities; and, whether they succeed or fail in healing the body, they are certain of leaving some comfort reflected in the soul.

(*To be continued.*)

